# Effects of internal low-dose irradiation from ^131^I on gene expression in normal tissues in Balb/c mice

**DOI:** 10.1186/2191-219X-1-29

**Published:** 2011-11-28

**Authors:** Emil Schüler, Toshima Z Parris, Nils Rudqvist, Khalil Helou, Eva Forssell-Aronsson

**Affiliations:** 1 Department of Radiation Physics, Institute of Clinical Sciences, Sahlgrenska Cancer Center, Sahlgrenska Academy at the University of Gothenburg, Sahlgrenska University hospital, Gothenburg, 413 45, Sweden; 2 Department of Oncology, Institute of Clinical Sciences, Sahlgrenska Cancer Center, Sahlgrenska Academy at the University of Gothenburg, Sahlgrenska University hospital, Gothenburg, 413 45 Sweden

**Keywords:** gene expression, low absorbed dose, iodide-131, irradiation, radiobiology, normal tissue damage

## Abstract

**Background:**

The aim of this study was to investigate the global gene expression response of normal tissues following internal low absorbed dose irradiation of ^131^I.

**Methods:**

Balb/c mice were intravenously injected with 13 to 260 kBq of ^131^I and euthanized 24 h after injection. Kidneys, liver, lungs, and spleen were surgically removed. The absorbed dose to the tissues was 0.1 to 9.7 mGy. Total RNA was extracted, and Illumina MouseRef-8 Whole-Genome Expression BeadChips (Illumina, Inc., San Diego, California, USA) were used to compare the gene expression of the irradiated tissues to that of non-irradiated controls. The Benjamini-Hochberg method was used to determine differentially expressed transcripts and control for false discovery rate. Only transcripts with a modulation of 1.5-fold or higher, either positively or negatively regulated, were included in the analysis.

**Results:**

The number of transcripts affected ranged from 260 in the kidney cortex to 857 in the lungs. The majority of the affected transcripts were specific for the different absorbed doses delivered, and few transcripts were shared between the different tissues investigated. The response of the transcripts affected at all dose levels was generally found to be independent of dose, and only a few transcripts showed increasing or decreasing regulation with increasing absorbed dose. Few biological processes were affected at all absorbed dose levels studied or in all tissues studied. The types of biological processes affected were clearly tissue-dependent. Immune response was the only biological process affected in all tissues, and processes affected in more than three tissues were primarily associated with the response to stimuli and metabolism.

**Conclusion:**

Despite the low absorbed doses delivered to the tissues investigated, a surprisingly strong response was observed. Affected biological processes were primarily associated with the normal function of the tissues, and only small deviations from the normal metabolic activity in the tissues were induced.

## Background

The biological effects of low absorbed doses and dose rates of ionizing radiation on normal tissue are today subjected to intense research and discussion. The most detailed knowledge of these effects comes from epidemiological studies based on data from A-bomb survivors and other populations exposed to ionizing radiation [1,2]. These data are, to a great extent, composed of high-dose and dose-rate exposures with mixed radiation types and inherent uncertainties in dosimetry. The current risk assessment used for radiation protection assumes that low-dose and low-dose-rate exposures result in the same risk per unit absorbed dose or effective dose compared to high-dose exposures (LNT model) [3-5].

Gene expression analysis using microarray technology can provide a comprehensive view of the biological effects of low doses of ionizing radiation. By studying cellular responses at the gene expression level, it may be possible to elucidate the mechanisms of radiation on normal tissues and identify genes linked to specific endpoints [6]. The impact of radiation on gene expression has predominantly been studied *in vitro*, possibly due to easier experimental conditions, e.g., one cell type, and better defined spatial and temporal exposures. However, *in vivo *studies are needed to elucidate the response of radiation on the different tissues and organs of the entire organism.

Few *in vivo *studies have been published with an analysis of gene expression alterations in tissues externally exposed by ionizing radiation and even fewer studies, using internal irradiation. The response in the brain tissue after an external acute high-dose irradiation (X-ray and gamma irradiation, 2 to 20 Gy) has been studied in mice [7,8]. The results showed an increasing number of modulated genes with the absorbed dose, and a peak in the number of upregulated transcripts with the dose was seen at 10 Gy after 5 h. A peak in the number of regulated transcripts was also found at 1 to 5 h after irradiation, however, with few genes in common between the different time points.

*In vivo *studies on the mouse liver with low-dose-rate irradiation showed results indicating a distinction between high- and low-dose exposures [9], which support the results found by Taki et al. in the mouse kidney [10]. Others have also investigated the effect on the mouse kidney with varying experimental protocols and results [8,11].

Iodine-131 [^131^I] is part of the uranium decay scheme and may be released into the environment by a nuclear accident. After the Chernobyl accident, the major cause of cancer in the affected areas was childhood thyroid cancer due to exposure mainly from ^131^I [12]. ^131^I is a radionuclide of interest in many applications. If introduced into the body, ^131^I is accumulated in the thyroid and to some extent, in the other organs [13,14]. Due to its biological, chemical, and physical properties, ^131^I is widely used in various diagnostic examinations as well as in radionuclide therapy of many different kinds of disorders [15-18].

The aim of this study was to investigate the effects of an internal exposure of ^131^I of low absorbed doses on the gene expression patterns in normal tissues in mice.

## Methods

### Irradiation

Female inbred BALB/c mice (Charles River, Salzfeld, Germany) were divided into four groups with two animals in each group. ^131^I in the form of sodium iodide (GE Health Care, Braunschweig, Germany) was diluted in phosphate-buffered saline (pH 7). Mice in three of the four groups were intravenously injected in the tail vein with 13, 130, and 260 kBq ^131^I, respectively, while the mice in the control group did not receive any injection. The animals had access to water and standard mouse food *ad libitum*. The experimental protocol was approved by the Ethical Committee on Animal Experiments in Gothenburg, Sweden.

The animals were euthanized 24 h after injection by pentobarbitalnatrium, and the kidneys, liver, lungs, and spleen were surgically removed. Tissue samples were immediately flash-frozen using liquid nitrogen and stored at -80°C until further analysis.

### Dosimetry

The absorbed dose to the different tissues investigated was calculated according to the Medical Internal Radiation Dose [MIRD] formalism [19]:

$${\bar{D}}_{\text{tissue}}={Ã}_{\text{tissue}}\times \sum n_{\text{i}}E_{\text{i}}\times \phi_{\text{i}}∕m_{\text{tissue}},$$

where *Ã*_tissue _is the cumulated activity during 24 h in the tissue investigated; *n*_i _is the probability that radiation, i, with the energy, *E*_i_, will be emitted per decay; *ϕ*_i _is the absorbed fraction of radiation, i; and *m*_tissue _is the mass of the tissue investigated. Only the contribution from the electrons emitted was included. Data for *Ã*, *n*_i_, *E*_i_, and *ϕ*_i _were found in the literature (Table 1) [20-22]. Briefly, the cumulated activity was determined from the biodistribution data from the same type of mice, assuming similar biokinetics irrespective of the activity administered (in the range studied), determined 4, 12, and 24 h after injection of ^131^I [22]. A monoexponential curve was fitted to the time-activity-concentration data and integrated over 24 h. The estimated absorbed dose in the tissues studied for the three groups is presented in Table 1.

**Table 1:** Dosimetric estimation

	Kidneys	Liver	Lungs	Spleen	Reference
Cumulated activity (*Ã*) (kBq·s)	161544	313027	217091	49087	Lundh et al. [22]
Energy per decay (*n*_i _× *E*_i_) (keV)	190	190	190	190	MIRD [21]
Absorbed fraction (*ϕ*_i_)	0.919	0.954	0.85	0.854	Flynn et al. [20]
Mass (g)	0.34	1.2	0.15	0.079	
*D *(13 kBq) (mGy)	0.17	0.10	0.49	0.21	
*D *(130 kBq) (mGy)	1.7	0.98	4.9	2.1	
*D *(260 kBq) (mGy)	3.5	2.0	9.7	4.2	

Values used for the absorbed dose calculation, *Ã*, *n*_i _× *E*_i _, and *ϕ*_i_, are given with references, together with the mass of the organs. The estimated absorbed doses, *D*, delivered to the different tissues from 13, 130, and 260 kBq ^131^I are shown.

### Gene expression analysis

The kidney cortex and medulla were separated. Fresh frozen tissue samples were pooled within the groups and homogenized using the Mikro-Dismembrator S ball mill (Sartorius Stedim Biotech, Aubagne Cedex, France). Total RNA was extracted using the RNeasy Lipid Tissue Mini Kit (Qiagen, Hilden, Germany) according to the manufacturer's instructions. RNA integrity was assessed using RNA 6000 Nano LabChip Kit with Agilent 2100 Bioanalyzer (Agilent Technologies, Santa Clara, CA, USA). Samples with RNA Integrity Number values above 6.0 were selected for further analysis.

The RNA samples were processed at the Swegene Center for Integrative Biology at Lund University. Hybridizations were performed on Illumina MouseRef-8 Whole-Genome Expression BeadChips (Illumina, Inc., San Diego, California, USA), containing 25,697 probes. Three independent hybridizations were performed on each sample to study technical variability. Images were acquired with the Illumina BeadArray Reader scanner and analyzed with the BeadScan 3.5.31.17122 image analysis software (Illumina, Inc., San Diego, California, USA).

### Data processing and statistical analysis

The web-based BioArray Software Environment system (BioArray Solutions, Ltd., Warren, NJ, USA) was used for data preprocessing and quantile normalization of the raw signal intensities, according to the recommendations given by Illumina. Further analysis was conducted using Nexus Expression 2.0 (BioDiscovery, El Segundo, CA, USA) using log2-transformed, normalized expression values and a variance filter.

The Benjamini-Hochberg method was used to control the false discovery rate [23]. Differential gene expression (at least 1.5-fold change) was deemed statistically significant if the *p *value after adjustment for multiple testing with the Benjamini-Hochberg method was lower than 0.01.

Affected biological processes were determined by identifying gene sets associated with different Gene Ontology [GO] terms. A *p *value cutoff of 0.05 was used. The GO data was further categorized into seven parental biological processes: metabolic processes, transport, cellular processes, system processes, developmental processes, immune response, and response to stimulus and stress. Gene expression data discussed in this publication have been deposited in NCBI's Gene Expression Omnibus [GEO:GSE32014].

### Quantitative real-time PCR

Seven genes (*Dao1 *in the kidney cortex and medulla, *Asprv1 *and *Ltf *in the lung, *Cfd *and *Lcn2 *in the spleen, and *Cyba *and *Cyb5r3 *in the liver) were selected from the gene list of significantly differentially expressed genes and analyzed using RT-PCR with predesigned TaqMan assays (Applied Biosystems, Carlsbad, CA, USA). Another three genes (*B2m*, *Gusb*, *Ywhaz*) with homogenous expression throughout the arrays were used for normalization. All reactions were performed on the cDNA synthesized from the same RNA extraction as the microarray experiments using SuperScript™ III First-Strand Synthesis SuperMix (Invitrogen, Carlsbad, CA, USA). Quantification was performed by the standard curve method. All samples were normalized by calculating the geometric mean of the three endogenous controls. The correlation between the two methods was calculated using the Pearson correlation coefficient.

## Results

### Dosimetry

The absorbed doses delivered to the different tissues investigated are presented in Table 1. The lowest and highest absorbed doses were received by the liver and lungs: 0.10 to 2.0 mGy and 0.49 to 9.7 mGy, respectively.

### Differential gene expression after irradiation

The number of regulated transcripts observed in the different tissues varied from 260 in the kidney cortex to 857 in the lung (Table 2). The number of regulated transcripts was thus the lowest for the kidneys, which is higher in the kidney medullary tissue than in the kidney cortex. Generally, upregulation was more prevalent in the analyzed specimens. In the spleen and lungs, about 70% of the regulated transcripts were upregulated. The liver revealed slightly lower values (around 60%). The kidney cortex showed the lowest fraction of upregulated transcripts at the middle absorbed dose but showed high values at the lowest and highest absorbed doses, while downregulation was more frequent for the kidney medulla. In the liver and lungs, the fraction of upregulated transcripts increased with the absorbed dose.

**Table 2:** Total number of regulated transcripts in ^131^I irradiated tissues

		Number of transcripts regulated per injected activity					
	Total number of transcripts regulated	13 kBq ^131^I		130 kBq ^131^I		260 kBq ^131^I	
Kidney medulla	423	160	↑50 (31%)	158	↑65 (41%)	208	↑65 (31%)
			↓110 (69%)		↓93 (59%)		↓143 (69%)
Kidney cortex	260	154	↑87 (56%)	85	↑30 (35%)	93	↑60 (65%)
			↓67 (44%)		↓55 (65%)		↓33 (35%)
Liver	738	417	↑250 (60%)	427	↑264 (62%)	455	↑292 (64%)
			↓167(40%)		↓163(38%)		↓163(36%)
Lung	857	320	↑149(47%)	113	↑82(73%)	596	↑475(80%)
			↓171(53%)		↓31(27%)		↓121(20%)
Spleen	607	240	↑158(66%)	306	↑240(78%)	238	↑176(74%)
			↓82(34%)		↓66(22%)		↓62(26%)

Data on changes in gene expression after i.v. injection of 13, 130, or 260 kBq. The total number of transcripts regulated in the tissues investigated is given together with the number of up- (arrows pointing up) and downregulated (arrows pointing down) transcripts given as the total number and percentage (in parentheses).

**Figure 1:**
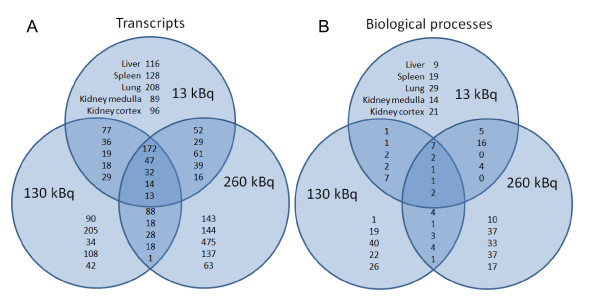
**Regulated transcripts and modulated biological processes**. Venn-diagram presenting the distribution of (**A**) the regulated transcripts and (**B**) the modulated biological processes between the different groups. Data for kidney cortex, kidney medulla, liver, lung, and spleen are shown. In general, more regulated transcripts and affected biological processes were specific for the different groups. In contrast, a more shared pattern of gene regulation for all three ^131^I activity levels was observed in the liver.

A clear distinction of regulated transcripts with absorbed dose could be seen in the different tissues (Figure 1A). In general, most regulated transcripts were specific for the different dose levels where few transcripts were affected at more than one dose level. Liver cells had the highest number of affected transcripts in common for all absorbed doses. A weak specific biological response (number of affected transcripts) was observed in the lung after 130 kBq of injected activity, IA, with a pronounced response at 13 and 260 kBq IA (34 vs. 208 and 475 regulated transcripts, respectively).

The most strongly affected gene found in the study was *Lor *in the lung (62 fold change) (Table 3). Overall, the lung had the strongest modulated transcripts with several transcripts revealing a power of regulation above 50 in fold change, all of which were upregulated. Negatively regulated transcripts in the lung revealed a much lower power of regulation. The strongest regulated genes in the kidney cortex and medulla, spleen, and liver were *Dao1*, *Cfd*, and OTTMUSG00000007485 transcript, respectively. All of these transcripts had a power of regulation above 8.

**Table 3:** Strongest modulated transcripts.

	Liver		Spleen		Lung		Kidney cortex		Kidney medulla	
	Up	Down	Up	Down	Up	Down	Up	Down	Up	Down
13 kBq ^131^I	*Mpo *(3.1)	*Slc25a25 *(-4.3)	-	*Cfd *(-9.2)	-	*Csrp3 *(-4.2)	*Fga *(4.0)	*Sgk1 *(-2.2)	*Slco4a1 *(2.9)	*Angptl4 *(-2.8)
	*Egr1 *(4.5)	*Ccrn4l *(-3.8)		*Cxcr4 *(-2.5)		*Mb *(-4.5)	*Fgg *(3.6)	*Gadd45g *(-2.7)	*Ly6f *(3.0)	
	*Lcn2 *(7.1)	*Coq10b *(-3.3)		*Cyp2e1 *(-2.7)		*Myh6 *(-4.9)	*Gdpd3 *(3.8)	*Angptl4 *(-2.8)	*Gdpd3 *(3.4)	
	*Ltf *(4.3)	*G6pc *(-3.2)		*Ddit4 *(-3.4)		*Myl1 *(-4.7)	*Ly6f *(3.5)			
	*Orm2 *(5.4)	*LOC620807 *(-3.1)		*Errfi1 *(-2.9)		*Myl4 *(-8.0)				
	*Prtn3 *(5.0)	*Mup1 *(-3.5)		*Tsc22d3 *(-3.0)		*Plunc *(-5.3)				
	*S100a8 *(4.6)	*Mup2 *(-3.8)				*Scgb3a1 *(-4.4)				
		*Pck1 *(-3.1)				*Sln *(-5.0)				
		*OTTMUSG00000007485* (-13)				*Tnnc1 *(-4.5)				
						*Mybphl *(-3.7)				
130 kBq ^131^I	*Lyz2 *(3.5)	-	*Mpo *(5.1)	*Slpi *(-2.4)	-	-	*Egr1 *(4.0)	*Clec2d *(-2.3)	*S100a8 *(3.3)	*Serpina1b *(-2.7)
	*S100a9 *(3.4)		*Lcn2 *(5.6)					*Gbp1 *(-2.5)		*Gbp1 *(-2.7)
			*Cd177 *(5.8)					*Igtp *(-2.5)		*Psca *(-3.1)
			*Chi3l3 *(7.0)					*Scd1 *(-2.8)		*Cfd *(-3.1)
			*Ctsg *(5.7)					*Adipoq *(-4.8)		*Cldn11 *(-3.3)
			*Prtn3 *(5.7)					*Car3 *(-5.7)		
								*Cfd *(-11.3)		
260 kBq ^131^I	*Hp *(4.2)	*Clec2d *(-3.1)	*Arg1 *(8.1)	*Ccl21b *(-2.8)	*Acta1 *(56)	-	*AU018778 *(6.3)	-	*Cyp4a12a *(2.9)	*Abcc3 *(-2.7)
			*Cxcl9 *(6.3)	*Ccl21c *(-2.5)	*Crct1 *(32)		*Cryab *(4.0)		*Cyp7b1 *(3.0)	*Akr1c12 *(-2.8)
			*Timp1 *(6.0)	*LOC100041504 *(-2.5)	*Krt13 *(50)		*Cyp2d9 *(5.0)		*Cyp2e1 *(3.2)	*Ly6f *(-3.6)
			*LOC100048556 *(6.3)		*Krtdap *(59)		*Dao1 *(11)		*Inmt *(3.8)	*Ddx6 *(-3.9)
					*Lce3c *(27)		*Inmt *(4.0)		*Cyp2d9 *(5.0)	
					*Lce3f *(38)				*Dao1 *(8.6)	
					*Lor *(62)					
					*Myh8 *(59)					
					*Rptn *(26)					

Ten most strongly up- and down-regulated transcripts in the different tissues investigated. Numbers in parenthesis indicate the fold change.

To identify transcripts regulated in two or more tissue types, tissues samples with similar absorbed dose were compared. The selected absorbed doses were 1.7, 2.0, 4.9, and 2.1 mGy for the kidneys, liver, lungs, and spleen, respectively. Few regulated transcripts were common for the different tissues (Table 4). No single transcript was regulated in all five tissue types. Among the transcripts regulated in more than one tissue, upregulation was more prevalent. In general, these transcripts were primarily associated with response to stimuli, immune response, metabolism, and transport. In addition, transcripts associated with cell cycle regulation and cell death were also identified. The spleen and lung had the highest number of modulated transcripts in common. Of these, three times more transcripts were up-regulated than down-regulated. In addition, several transcripts revealed opposite modulation between tissues.

**Table 4:** Transcripts in common between two or more tissues

Liver	Spleen	Lung	Cortex	Medulla	Number	Genes in common	Comment
↑	↑				16	*Ela2*, *Orm1*, *Ngp*, *Anxa3*, *Mpo*, *Lrg1*, *Hp*, *Hp*, *Lcn2*, *Ltf*, *Prtn3*, *Camp*, *Lbp*, *S100a9*, *Actb*, *Ear4 *	Response to stimulus; metabolism, transport
↓	↑				1	*Aatk*	Cell death
↑	↓				5	*H2-Ab1*, *Hspd1*, *Serpina3h*, *Hspa8*, *Creld2 *	Response to stimulus; immune response
↓		↑			1	*LOC100048480*	
↑		↓			4	*Serpina3g*, *EG667977*, *Hspa8*, *H2-Q8*	Response to stimulus; immune response
↑		↑			9	*Ngp*, *Mpo*, *Chac1*, *S100a8*, *Ltf*, *Camp*, *Lbp*, *S100a9*, *Actb*	Response to stimulus; transport
↑			↑		3	*Lyz2*, *S100a8*, *Cxcl1*	Response to stimulus; immune response
↓			↓		7	*Hmgcs2*, *Gja1*, *Baat*, *LOC100048480*, *Clec2d*, *Mmd*, *Cyb5*	Immune response; developmental process; metabolism
↓			↑		5	*Cxcl9*, *Cd74*, *Thrsp*, *Car3*, *H2-Q5*	Immune response; metabolism
↓				↓	1	*Sgk1*	
↓				↑	2	*Hrsp12*, *LOC100048480*	
↑				↓	6	*Cxcl9*, *Cd74*, *Car3*, *Lcn2*, *Serpina3g*, *H2-K1*	Immune response; metabolism; transport
↑				↑	11	*Cyp2d26*, *Insig1*, *Chrna4*, *Cyp4a12a*, *Rnase4*, *S100a8*, *S100a9*, *Chrna4*, *Ang*, *Hmox2*, *Pdhb*	Response to stimulus; transport
	↓	↓			7	*Serpina1d*, *Dnajb1*, *Serpina1b*, *Errfi1*, *Hsp105*, *Angptl4*, *Hspa8*	Response to stimulus
	↓	↑			2	*Esm1*, *Cfd*	Immune response
	↑	↑			23	*Ifitm6*, *Rsad2*, *Ngp*, *Stfa2*, *Pglyrp1*, *Mpo*, *1100001G20Rik*, *Retnlg*, *Asprv1*, *Mmp9*, *Chi3l3*, *Arl2bp*, *Arl2bp*, *Ltf*, *Camp*, *Lbp*, *Cd177*, *S100a9*, *Actb*, *Stfa1*, *EG433016*, *Chi3l3*, *Chi3l3*	Response to stimulus; cellular process; transport
	↓		↓		4	*Scd1*, *LOC668837*, *Angptl4*, *Cfd*	Immune response; metabolism
	↓		↑		1	*Napsa*	
	↑		↓		1	*Spc25*	Cell cycle regulation
	↑		↑		3	*Arl2bp*, *Arl2bp*, *Hdc*	Cell cycle regulation
	↓			↓	7	*Serpina1d*, *Slpi*, *Akr1b3*, *Serpina1b*, *Stbd1*, *Angptl4*, *Cfd*	Response to stimulus; immune response; metabolism
	↑			↓	6	*Klf5*, *4930519N13Rik*, *Lcn2*, *S100a6*, *Hdc*, *AA467197*	Cell cycle regulation; transport
	↑			↑	2	*Arl2bp*, *S100a9*	Cell cycle regulation
		↓	↓		4	*Gbp1*, *Iigp2*, *Igtp*, *Angptl4*	Immune response
		↑	↓		3	*LOC100048480*, *Adipoq*, *Cfd*	Immune response; metabolism
		↑	↑		6	*Junb*, *Egr1*, *S100a8*, *Arl2bp*, *Arl2bp*, *Cyp2a5*	Response to stimulus; metabolism
		↓		↓	10	*Serpina1b*, *Serpina1d*, *Gbp1*, *Iigp2*, *Igtp*, *Cdkn1a*, *Serpina1b*, *Serpina3g*, *Angptl4*, *Hspa1a*	Response to stimulus; immune response; cell cycle regulation
		↑		↓	2	*Adipoq*, *Cfd*	Immune response; metabolism
		↑		↑	6	*Pdrg1*, *LOC100048480*, *Egr1*, *S100a8*, *Arl2bp*, *S100a9*	Response to stimulus
			↓	↓	13	*Cidea*, *Cxcl9*, *Cd74*, *Gbp1*, *H2-DMb1*, *Iigp2*, *Igtp*, *Car3*, *Adipoq*, *Psmb10*, *Angptl4*, *Cfd*, *Gbp2 *	Immune response; metabolism
			↓	↑	1	*LOC100048480*	
			↑	↓	1	*Hdc*	
			↑	↑	7	*Hsd3b2*, *Cyp24a1*, *Egr1*, *S100a8*, *Arl2bp*, *Pcsk9*, *Dao1 *	Response to stimulus; metabolism
↑	↓	↓			1	*Hspa8*	Response to stimulus
↑	↑	↑			7	*Ngp*, *Mpo*, *Ltf*, *Camp*, *Lbp*, *S100a9*, *Actb*	Response to stimulus; transport
↑	↑			↓	1	*Lcn2*	Transport
↑	↑			↑	1	*S100a9*	
↓		↑	↓		1	*LOC100048480*	
↑		↑	↑		1	*S100a8*	Response to stimulus
↓		↑		↑	1	*LOC100048480*	
↑		↓		↓	1	*Serpina3g*	Immune response
↑		↑		↑	2	*S100a8*, *S100a9*	Response to stimulus
↓			↓	↑	1	*LOC100048480*	
↑			↓	↓	3	*Cxcl9*, *Cd74*, *Car3*	Immune response; metabolism
↑			↑	↑	1	*S100a8*	Response to stimulus
	↓	↓	↓		1	*Angptl4*	
	↓	↑	↓		1	*Cfd*	Immune response
	↑	↑	↑		2	*Arl2bp*, *Arl2bp*	
	↓	↓		↓	3	*Serpina1d*, *Serpina1b*, *Angptl4*	Response to stimulus
	↑	↑		↑	2	*Arl2bp*, *S100a9*	
	↓		↓	↓	2	*Angptl4*, *Cfd*	Immune response
	↑		↑	↓	1	*Hdc *	
	↑		↑	↑	1	*Arl2bp*	
		↓	↓	↓	4	*Gbp1*, *Iigp2*, *Igtp*, *Angptl4*	Immune response
		↑	↓	↓	2	*Adipoq*, *Cfd*	Immune response
		↑	↓	↑	1	*LOC100048480*	
		↑	↑	↑	3	*Egr1*, *S100a8 Arl2bp*	Response to stimulus
↓		↑	↓	↑	1	*LOC100048480*	
↑		↑	↑	↑	1	*S100a8*	Response to stimulus
↑	↑	↑		↑	1	*S100a9*	
	↓	↓	↓	↓	1	*Angptl4*	
	↓	↑	↓	↓	1	*Cfd*	Immune response
	↑	↑	↑	↑	1	*Arl2bp*	

Comparison was conducted with the aim of keeping the absorbed dose to the different tissues constant. The absorbed doses for the different tissues were 1.7, 2.0, 4.9, and 2.1 mGy for the kidney, liver, lung, and spleen, respectively. Arrows that are pointing up denote upregulation, while arrows that are pointing down denote downregulation.

**Figure 2:**
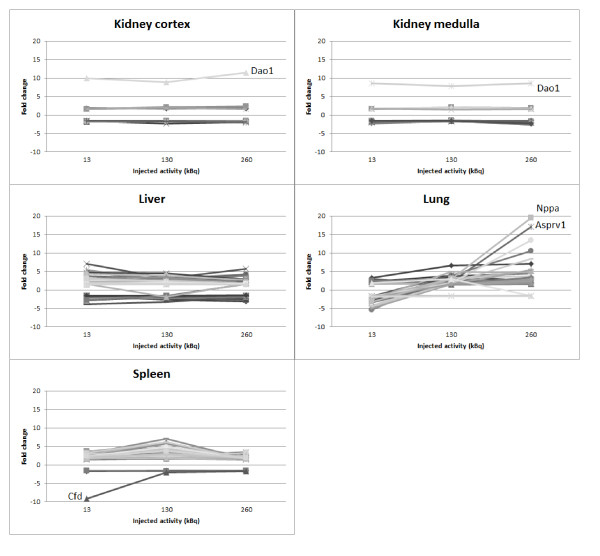
**Dose-response relationship for the transcripts found to be regulated at all absorbed dose levels**.

The dose-response relationship for each tissue type was studied for the transcripts regulated at all dose levels (Figure 2). The dose-response relationship found in the kidney cortex and the kidney medulla was similar, and most transcripts were either up- or downregulated by a factor of 2 at all dose levels. The only exception was the transcript associated with the *Dao1 *gene whose expression was markedly stronger compared to the other regulated transcripts. In the liver and kidney tissues, the majority of the regulated transcripts showed little difference in response between the different absorbed doses. In contrast, the lung showed a strong variation in response between the different absorbed dose levels, where a high percentage of the regulated transcripts were downregulated at 13 kBq IA while upregulated at 130 and 260 kBq IA, e.g., *Nppa*, *Cfd*, *Plunc*, and *Mb*. In both lung and spleen, few transcripts were consistently downregulated.

Differences in gene expression between the groups were verified using quantitative polymerase chain reaction [QPCR]. The genes were assessed for all absorbed dose levels in the tissues, and the QPCR and microarray data were strongly correlated for the genes *Asprv1*, *Ltf*, *Cfd*, *Cyba*, and *Cyb5r3 *(*r *> 0.86) (*Dao1 *was excluded due to technical issues). However, no correlation was found between the *Lcn2 *gene expression using microarray and QPCR analysis.

### Biological processes

Shared and specific biological processes were detected after irradiation of the analyzed tissues. The number of affected biological processes ranged from 37 in the liver to 108 in the lung (Figure 1B) [see Additional file 1]. In general, affected dose-specific biological processes were more frequent compared to the affected processes observed at all absorbed dose levels. In the lung, which had the highest number of modulated biological processes (108 processes), only 6 processes were detected at two or more absorbed dose levels. This can be compared to the liver, which had the lowest number of modulated biological processes (37 processes), where as many as 17 processes were detected at two or more absorbed dose levels.

In total, 70 biological processes were affected in two or more tissue types (Table 5). The highest number of affected processes in common for two tissue types was observed for the kidney cortex-lung tissue combination and the kidney cortex-kidney medulla tissue combination. Both of these tissue combinations had 20 processes in common, which were closely followed by the kidney medulla-lung tissue combination with 18 commonly affected processes. The kidney medulla-liver and liver-spleen tissue combinations had the fewest number of biological processes in common with only three and five processes, respectively. Interestingly, immune response was the only biological process in common for all investigated tissues.

**Table 5:** Common biological processes

Tissue combination	Biological process
Kidney cortex-kidney medulla	Amiloride transport
	Amino acid transport
	Bone remodeling
	Canalicular bile acid transport
	Choline metabolism
	Negative regulation of cell adhesion
	Negative regulation of enzyme activity
	Positive regulation of actin filament polymerization
	Protection from natural killer cell mediated cytotoxicity
	Regulation of hormone secretion
	Transport
Kidney cortex-liver	Acetyl-CoA metabolism
	Cytolysis
	Response to sterol depletion
	Retinoid metabolism
	Steroid biosynthesis
	Thermoregulation
Kidney cortex-lung	Cellular response to starvation
	Cytoskeleton organization and biogenesis
	Fatty acid oxidation
	Patterning of blood vessels
	Positive regulation of glucose import
	Positive regulation of lipid metabolism
	Protein folding
	Regulation of transcription from RNA polymerase II promoter
Kidney cortex-spleen	Regulation of axon extension
	Regulation of neuronal synaptic plasticity
	Response to oxidative stress
	Ubiquitin-dependent protein catabolism
Kidney medulla-liver	Digestion
Kidney medulla-lung	Cell migration
	Cell-matrix adhesion
	Cellular defense response
	Positive regulation of angiogenesis
	Positive regulation of neurotransmitter secretion
	Regulation of locomotion
	Regulation of long-term neuronal synaptic plasticity
	Response to hypoxia
	Synaptic vesicle transport
Kidney medulla-spleen	Cartilage condensation
	Central nervous system development
	Complement activation; alternative pathway
	Neuropeptide signaling pathway
	Positive regulation of small GTPase mediated signal transduction
	Response to nutrient
	*S*-adenosylmethionine biosynthesis
Liver-lung	Embryonic heart tube development
	Fatty acid metabolism
	Metabolism
Liver-spleen	Negative regulation of signal transduction
	Regulation of cell growth
Lung-spleen	Iron ion homeostasis
	Peptidoglycan metabolism
	Response to biotic stimulus
Kidney cortex-kidney medulla-lung	Negative regulation of apoptosis
Kidney cortex-kidney medulla-spleen	Defense response
Kidney cortex-liver-lung	Acute-phase response
	Complement activation
	Lipid metabolism
Kidney cortex-lung-spleen	Response to glucose stimulus
Kidney medulla-lung-spleen	Positive regulation of non-apoptotic programmed cell death
Liver-lung-spleen	Response to heat
	Response to unfolded protein
Kidney cortex-kidney medulla-liver-lung	Electron transport
Kidney cortex-kidney medulla-lung-spleen	Inflammatory response
	Negative regulation of gluconeogenesis
	Negative regulation of lipoprotein lipase activity
	Positive regulation of fatty acid metabolism
	Positive regulation of signal transduction
Kidney cortex-kidney medulla-liver-lung-spleen	Immune response

Biological processes affected in two or more tissues independent of the absorbed dose. All processes had a *p *value < 0.05. Additional information is presented in Additional file 1.

The biological processes modulated in the investigated tissues were primarily associated with metabolism, transport, immune response, and response to stimuli, as well as cellular, system, and developmental processes (Table 6). Several of these parental biological processes were highly tissue-specific as a distinctive difference in the proportion of over-represented biological processes was observed between the different tissues. The kidneys and lungs had a strong association with transport, while the liver had a strong association with metabolism. Cellular processes were primarily associated with the spleen and kidney medulla; system processes were strongly associated with the lungs, and immune response was strongly associated with the spleen. Processes which had more than one transcript associated with it were included in this categorization.

**Table 6:** Parental biological processes

	Kidney cortex			Kidney medulla			Liver			Lung			Spleen		
	13 kBq	130 kBq	260 kBq	13 kBq	130 kBq	260 kBq	13 kBq	130 kBq	260 kBq	13 kBq	130 kBq	260 kBq	13 kBq	130 kBq	260 kBq
Metabolism	27% (3)	38% (9)	34% (5)	11% (1)		8% (1)	59% (13)	85% (11)	54% (14)	9% (3)	27% (8)	57% (21)	46% (6)	30% (7)	30% (7)
Transport	27% (3)	4% (1)		11% (1)	18% (2)						17% (5)	11% (4)	15% (2)		4% (1)
Cellular process	9% (1)	13% (3)	13% (2)	33% (3)	9% (1)	42% (5)	5% (1)	8% (1)	4% (1)	38% (12)	10% (3)	5% (2)		44% (10)	13% (3)
System process		4% (1)	7% (1)			8% (1)	9% (2)				17% (5)	16% (6)			
Developmental process	18% (2)	8% (2)	13% (2)	22% (2)	27% (3)	25% (3)	9% (2)	8% (1)	12% (3)	3% (1)	10% (3)	8% (3)			4% (1)
Immune response		13% (3)	13% (2)		18% (2)		9% (2)		12% (3)	22% (7)			15% (2)	17% (4)	9% (2)
Response to stimulus and stress	18% (2)	21% (5)	20% (3)	22% (2)	27% (3)	17% (2)	9% (2)		19% (5)	28% (9)	20% (6)	3% (1)	23% (3)	9% (2)	39% (9)

The fraction of affected biological processes in the investigated tissues, grouped into parental biological processes. The numbers are calculated as the fraction of affected processes per parental process and per injected activity divided by the total number of processes affected per injected activity and tissue. The number of biological processes is given in parenthesis.

## Discussion

In the present study, the effects of internal low-dose irradiation by ^131^I were investigated *in vivo*. Using gene expression microarray, differentially expressed transcripts were analyzed, and affected biological processes were investigated. A strong biological response was detected following the low absorbed doses delivered. Although low amounts of ^131^I were administered, a homogenous absorbed dose distribution in the tissues studied can be assumed. No difference in the absorbed doses delivered to the kidney cortex and medulla was assumed due to the long range beta particles emitted by ^131^I: an average continuous slowing down approximation [CSDA] range of 0.41 mm and a maximum CSDA range of up to 1 mm in water [24]. However, in organs with a higher concentration than the surrounding tissue, a lower absorbed dose in the outermost cells of the organ can be assumed [25].

The majority of studies on cellular response to irradiation have been performed using cell cultures, where it is possible to control several components, such as cell type and irradiation homogeneity. Few experiments and results are reported from *in vivo *models. Some reasons might be due to tedious animal handling, heterogeneity in absorbed dose, the mixture of cell types within the tissues, and effects related to the increased complexity of the system. In the present type of *in vivo *study, the tissue response should be different from the response observed *in vitro *because the systemic administration of ^131^I results in irradiation of all organs and tissues, although to various absorbed doses, and thus to systemic effects. In addition, cell communication and heterogeneity within and between tissues and organs make the cellular response *in vivo *more complex compared to the *in vitro *response. In this study, total RNA was extracted from whole tissue samples (kidney medulla, kidney cortex, liver, lungs, and spleen) containing heterogeneous cell populations. One major problem is, then, that weak or moderate modulation of transcripts present in a subpopulation of cells in an organ may become undetectable [10,11]. In the separation of the kidney medulla and cortex, contamination between the samples is unavoidable. However, distinct gene expression profiles were observed between these two tissues. In addition, Balb/c mice were used which are an inbred strain with an immunologic deficiency. The results presented in this study are therefore specific to this strain of mice. The differences in the response to irradiation have previously been reported between Balb/c and C57BL/6 mice after low-dose irradiation (0.2 Gy) to the liver [26]. A comparison between the two revealed 37 genes which were differentially expressed in both strains. Of these 37 genes, 14 showed similar expression patterns. The remaining genes were primarily involved in various signal transduction processes. However, key responses to radiation are highly probable to be similar between the different strains of mice. The highest number of affected transcripts was detected in the lungs, with a complex dependence on absorbed dose. The number of transcripts affected in the group injected with 130 kBq was lower (113) in comparison with the number of transcripts detected in the groups receiving 13 and 260 kBq (320 and 596, respectively). In contrast, a reverse relationship was observed in the number of affected biological processes since the group receiving 130 kBq had the highest number of affected biological processes. Interestingly, the groups receiving 13 and 260 kBq shared the largest number of transcripts, but with no affected biological processes in common. While the number of affected biological processes does not necessarily follow the distribution found in the number of regulated transcripts, the complexity of the distributions is noteworthy. In the lung and liver tissues, the fraction of upregulated transcripts increased with the absorbed dose, with the highest increase observed in the lung (from 47% to 80% compared to the increase from 60% to 64%). No such increase could be seen in the kidney tissues, while in the spleen, the fraction of upregulated transcripts increased from 66% to 78% between the groups receiving 13 and 130 kBq, followed by a decrease in the group that received the highest injected activity.

A closer examination of the dose-response relationships for transcripts regulated at all doses in a certain tissue type showed that few transcripts could potentially serve as biomarkers for the absorbed dose in the dose interval studied, i.e., showing a monotone increase or decrease in expression with the absorbed dose. The majority of the affected transcripts showed little or no difference in the response between the different absorbed dose levels. In the lung, a high percentage of the regulated transcripts showed a negative regulation at the lowest absorbed dose level and a positive regulation at the two higher absorbed dose levels. Transcripts associated with the *Cyp2a5*, *Mb*, *Sln*, *Scgb3a1*, and *Plunc *genes in the lungs and the *Clec2d*, *Wsb1*, *Mup4*, *Acaa2*, and *Mpo *genes in the liver showed a monotone increase or decrease in expression with the absorbed dose. An example of more extreme modulation between dose levels was demonstrated by the *Nppa *gene, which showed a negative regulation at the lowest dose level that transitioned to a positive regulation with a power of 3 and 20 at 130 and 260 kBq, respectively. An example of a weak regulation at the two lower absorbed dose levels followed by a strong regulation at the highest dose level was demonstrated by the *Asprv1 *gene (1.6, 2.1, and 17 at 13, 130, and 260 kBq, respectively). In the spleen, the reverse relationship could be seen where *Cfd *showed a nine-fold decrease in expression at the lowest absorbed dose level followed by an increase to about a two-fold decrease at the two higher absorbed dose levels. Whether these or other transcripts investigated could potentially prove to be good biomarkers for absorbed dose is still to be determined, and more studies are needed with a larger interval of absorbed doses, together with analyses using QPCR, immunohistochemistry, and Western blot to study the impact at the protein level.

The biological processes affected in the irradiated tissues were grouped according to seven parental biological processes (metabolism, transport, cellular processes, system processes, developmental processes, immune response, and response to stimuli and stress). The type of biological processes affected was, to a great extent, tissue-specific. However, immune response was affected in all tissues. It has been shown that radiation induces effects linked to the immune response and that these types of effects could be observed from hours up to several weeks after exposure [27]. Furthermore, in addition to a strong association to cellular processes in the spleen and kidney medulla, the effects on the spleen were primarily associated with cell cycle regulation (data not shown). Among the ten affected biological processes that were associated with cell cycle regulation, nine were detected in the spleen. However, the affected processes were closely linked to the normal functions of the investigated tissues, indicating that the specific effects from irradiation were low.

When comparing the biological processes affected in the different tissues, the kidney medulla-liver and liver-spleen tissue combinations had the fewest modulated processes in common. Both the difference in the types of cells which comprise the liver and spleen (hepatocytes, Kupffer cells, and fat-storing cells versus lymphocytes) and the function of these two organs (metabolic functions and detoxification versus immune defense and blood storage), which are very different in nature, may explain the presence of having few processes in common. However, both the liver and spleen are part of the mononuclear phagocyte system which should suggest a more similar response between the tissues. The question then is why some tissues had more affected biological processes in common. It has previously been stipulated that tissue-specific intracellular signaling pathways are responsible for the markedly different responses found in different tissues following irradiation and that signaling pathways inherently active would be used as a response to the induced stress [8]. This argument could explain why few transcripts and biological processes were affected in two or more tissue types after irradiation.

Iodide administered into the body is primarily accumulated in the thyroid gland by uptake into the thyrocytes and incorporation into the metabolically related thyroid hormones [28]. No control group with stable iodide in the same order as that of ^131^I was included in this study. We do not believe that such a control group would be of any value due to the high iodine concentration in the normal mouse chow. The amount of radioiodide administered in a mouse in the highest absorbed dose group is only one tenth of what each animal consumes in 1 day. Therefore, we believe that the effects obtained in the present study are mainly related to the exposure to ionizing radiation. The injections of ^131^I were done by a very experienced animal technician to reduce potential stress of the injected animals. Unfortunately, no injection was made in the control group. However, no general increased stress response was found, resulting in the common regulation of genes between the tissues and absorbed doses; only a few genes were common between the different absorbed dose levels. This fact also strengthens the interpretation that the differences found between the irradiated mice and controls were due to the ionizing radiation exposure.

Knowledge about the effects of internal irradiation on the whole genome gene expression on organisms *in vivo *is scarce. To our knowledge, no study has been published presenting radiobiological data at the low absorbed dose levels and dose rates used in the present study. However, two studies have presented results for the mouse kidney and liver at absorbed dose levels as low as 20 mGy after continuous external low-dose-rate irradiation for more than 400 days [9,10]. The results of these studies showed minimal response with less than six genes regulated in either of the studies. No similarity between our results and the results from these two studies was found either in the number of modulated genes or in the specific genes modulated. While the number of regulated transcripts were below six in these two studies, our results showed a much stronger response with 93, 208, and 455 modulated transcripts for the kidney cortex, kidney medulla, and liver, respectively, in the group injected with the highest ^131^I activity. The reason for these discrepancies would most probably be due to the large differences in the irradiation protocol between the two previous studies and the present study. The dose rate in the two earlier studies were between 0.029 and 0.032 μGy/min for the 20 mGy dose level, while in the present study, the mean dose rate was 2.4 and 1.4 μGy/min for the highest injected activity for the kidney and liver, respectively. Discrepancies are most likely also due to the differences in the time of irradiation and time after the start of irradiation. A previous study on human myeloid leukemia cells has shown a general decrease in the power of gene modulation with decreasing dose rate and that some genes showed a clear dose rate dependency while others did not, which further confirms the large differences seen [29]. Others have also investigated the biological effects after high- and low-dose-rate irradiation and found a great difference in the modulated genes, with 2,421 and 608 differentially regulated genes after high- and low-dose irradiation, respectively, in the thymus tissue [30]. Mice were irradiated with external irradiation with either 0.8 Gy/min or 0.7 mGy/h for 5.6 min and 268 days, respectively, up to a total absorbed dose of 4.5 Gy. The results showed a dramatic downregulation of the immune response in the high-dose-rate irradiated mice, together with an increasing risk of thymic lymphoma. A dose rate effect can also be assumed to be present within the results presented in the present study; the absorbed dose was delivered at varying dose rates with time (including effects of biokinetics and physical decay of the radionuclide) as well as with dose, which is an unavoidable consequence of using internal radiation emitters for exposure. A dose rate effect is most likely present, and it can be assumed that this effect has a higher impact with dose compared with time in this study due to the relatively long half-life of ^131^I.

## Conclusion

^131^I is a commonly used radionuclide in routine medicine both for diagnostics and for therapy. While the overall side effect (both acute and late effects) on normal tissues from high-dose exposures is relatively well known, the effects in the low-dose range is still to be explored. Notably, firm data on the risk of cancer development at low-dose irradiation are needed. The results from this study clearly demonstrate radiation-induced regulation of gene expression in the tissue types studied, already at these low absorbed dose levels. The biological response was to some extent tissue-specific, but some pathways affected by radiation were also detected in several tissue types. The data also indicate that only small deviations from the normal functions of the tissues were induced. However, the impact of these deviations is unknown, and further research is needed to evaluate late biological effects.

## Competing interests

The authors declare that they have no competing interests.

## Authors' contributions

All authors were involved in the design of the trial and took part in the interpretation of the data and in revising the manuscript. ES carried out the analysis of data and drafted the manuscript. NR carried out the animal trial. TP and ES carried out the extraction of total RNA. All authors read and approved the final manuscript. All authors have given their final approval of the version to be published.

## Supplementary Material

Additional file 1 **Additional information on the biological processes in the different tissue types**. A supplementary table consisting of additional data on the different biological processes in the different tissue types. Click here for file
